# A Clinical Report of Two Cases of Cryptogenic Brain Abscess and a Relevant Literature Review

**DOI:** 10.3389/fnins.2018.01054

**Published:** 2019-01-14

**Authors:** Wei Zhou, Xuefei Shao, Xiaochun Jiang

**Affiliations:** Department of Neurosurgery, Yijishan Hospital, Wannan Medical College, Wuhu, China

**Keywords:** brain abscess, cryptogenic, *Streptococcus*, diagnosis, treatment

## Abstract

Brain abscess, a severe intracranial infectious disease, refers to the parenchyma abscess caused by local infection or remote spread. Recently, advancements in modern medicine, especially the wide application of antimicrobial drugs, have contributed to the gradual decrease in the prevalence of this disease. However, cases of cryptogenic brain abscess that feature an unknown origin and atypical symptoms are rising. In this retrospective study, we report and analyze two cases of cryptogenic brain abscess. The first patient was a 30-year-old healthy man who was admitted to our hospital due to 1 week of headache and 3 days of headache aggravation, accompanied by nausea and vomiting. Head MRI shows a circular space-occupying as well as apparently enhanced DWI signals were observed in the right parietal lobe, and the ring wall manifested an apparent increase in signal intensity after enhancement. The patient was diagnosed as a brain abscess before operation and given craniotomy. The postoperative pathology confirmed brain abscess and recovered well after surgery. The second patient was a 45-year-old healthy woman who was hospitalized in a local hospital due to symptoms of headache and right limb weakness for 1 week. Head MRI shows a circular space-occupying lesion in the left basal ganglia, and the ring wall manifested an apparent increase in signal intensity after enhancement. The patient was suspected of glioma at the local hospital and was transferred to our hospital. Twelve hours after hospitalization, the patient was suspected of developing cerebral palsy and thus underwent emergency surgery including lesion resection in the left basal ganglia, resection of the polus temporalis, and a decompressive craniotomy. Postoperative pathology confirmed brain abscess. The patient was eventually conscious, but left the right limb hemiplegia. Hence, when a patient develops the classical triad of fever, headache, and focal neurologic deficits, the possibility of brain abscess should be investigated. Early diagnosis and treatment are crucial to minimize various complications and the number of deaths.

## Introduction

Brain abscess is a severe intracranial infectious disease that has a prevalence of 0.4–0.9 per 100,000 population (Nicolosi et al., 1991; Helweglarsen et al., 2012) as well as high disability and morality rates. Improvements in its diagnosis and treatment, along with advancements in imaging technologies (in particular, CT and MRI) have reduced the mortality rate of brain abscess from 40 to 10% (Brouwer et al., 2014). Brain abscess results from the invasion of an external infection into the brain tissue via a variety of routes such as haematogenous spread, direct spread, and traumatic infection. Its clinical manifestations primarily include acute infection, increased intracranial pressure, and brain focal signs. However, some patients with brain abscess carry insidious sources of infection that cannot be identified; these cases are termed “cryptogenic brain abscess.” As cryptogenic brain abscess lacks distinctive traits in its disease history, clinical symptom, and physical signs, it is prone to misdiagnosis and mistreatment, and it has a poor prognosis. The current study reports two cases of cryptogenic brain abscess. Both patients had a recent history of good physical health, with no record of otitis media, sinusitis, heart diseases, head injury, or recent infection. The two patients showed clinical differences in the lesion site and disease progression rate, but both underwent surgery. Both patients received adequate medication after surgery. The results are detailed below. The publication of this case report obtained written informed consent from two patients.

## Case Presentation

**FIGURE 1:**
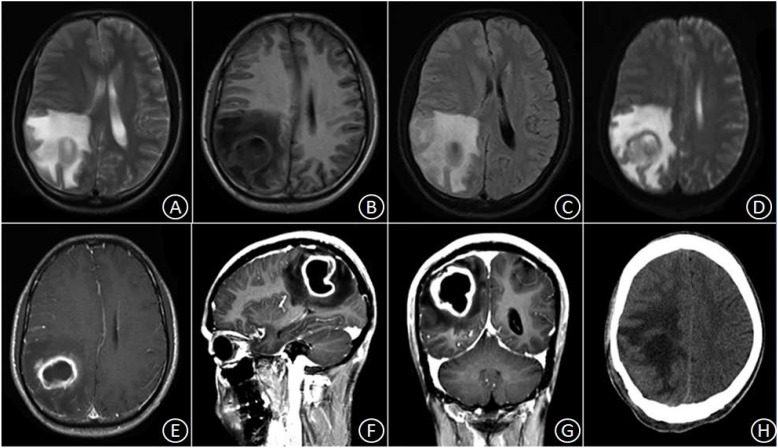
In the right parietal lobe, an irregularly circular space-occupying lesion was found with slightly long T1 and slightly long T2 signal shadows, in which sheet-like, long T1 and T2 signals were observed surrounded by large patches of oedema **(A–C)**. DWI signals were apparently enhanced, and the marginal ring wall appeared to have a relatively even thickness, which manifested as slightly short T1 and T2 signals **(D)**. After enhancement, the ring wall exhibited apparent signal intensification **(E–G)**. Three weeks after the surgery, the space-occupying lesion disappeared, but patches of oedema with blurred boundary remained **(H)**.

Patient No. 1 was a 30-year-old male with a history of good physical health; a diagnostic consultation did not identify any foci of infection or record of otitis media, sinusitis, or head trauma. His major complaints included 1 week of headache and 3 days of headache aggravation, accompanied by nausea and vomiting. The patient was hospitalized at our department with a body temperature of 36.0°C. The physical examination report conducted at the Department of Neurology showed that the patient had clear consciousness, was apathetic, had a Glasgow Coma Scale (GCS) score of 15, a soft neck, normal limb muscular strength, normal deep, and superficial sensations. After hospitalization, a complete blood count reported a white blood cell count of 8.4 × 10^9^/L (Normal reference range: 4.0–10.0 × 10ˆ9/L), 70.4% (Normal reference range: 50–75%) neutrophils, 20.1% (Normal reference range: 20–40%) lymphocytes, and C-reactive protein (CRP) level of 9.0 mg/L (Normal reference range: 0–10 mg/L). A cardiac echo reminded the heart that everything was normal. Head magnetic resonance imaging (MRI), including a plain scan and an enhancement scan, showed the following: (i) irregularly circular, slightly long, aberrant T1 and T2 signal shadows with sheet-like, long T1 and T2 signals as well as apparently enhanced diffusion-weighted imaging (DWI) signals were observed in the right parietal lobe; (ii) an edge ring wall with a relatively even thickness presented as slightly short T1 and T2 signals; (iii) the ring wall manifested an apparent increase in signal intensity after enhancement; (iv) the lesion was surrounded by large patches of T1 and T2 oedema shadow; (v) the adjacent ventricles and parenchyma displayed compression-resulted deformation; (vi) the midline structures were slightly shifted toward the left (Figures 1A–G). The patient received resection of the space-occupying lesion in the right parietal lobe. During the operation, a dark yellow and intact capsule (abscess wall) with brittle texture was visible. Because of its high internal pressure, the yellow, viscous pus was first aspirated to relieve the pressure before the capsule was completely excised. Some residual tissues did not have a clear boundary with the normal brain tissue. A pathological examination showed the presence of inflammatory exudates, necrosis, and granulations (Figures 3A,B). After the operation, the patient was provided with an empiric regimen of 1 g meropenem q8h and 1 g vancomycin q12h. A pus culture indicated the presence of *Viridans streptococci*, and a drug sensitivity test showed that the bacterium was sensitive to vancomycin; thus, the patient was treated with vancomycin 1 g q12h. After the operation, the patient had a low-grade fever that fluctuated around 37.5°C. After 1 week of treatment, his body temperature returned to normal. The complete blood count revealed the following: leukocyte count of 7.1 × 10^9^/L, 65.4% neutrophils, 24.5% lymphocytes, and CRP level of 8.2 mg/L. As such, the patient continued the vancomycin regimen for 2 additional weeks, after which he displayed a remission of the headache and showed normal limb strength. A head computed tomography (CT) scan showed that the space-occupying lesion in the right parietal lobe had disappeared, the surrounding area had large patches of low-density shadows with a blurred boundary, and the midline structures displayed local left-shift (Figure 1H). The patient was discharged. A physical examination showed that the patient had a normal body temperature, clear consciousness, good articulation, and normal limb muscular strength.

**FIGURE 2:**
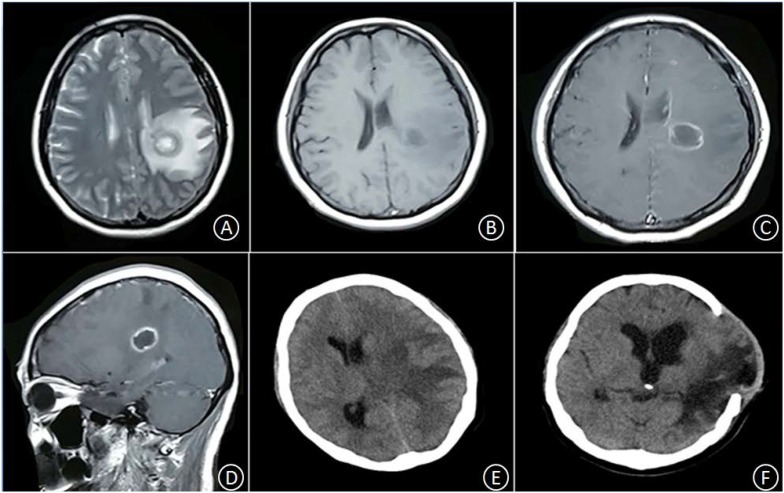
In the left basal ganglia area, an aberrant, circular space-occupying lesion with long T1 and long T2 signal shadows was observed. Its ring wall had relatively even thickness, manifesting as slightly short T1 and T2 signal shadows **(A,B)**. The lesion-surrounding area contained patches of apparent oedema; the left ventricle displayed slight compression-resulted deformation; and the midline structures displayed minor rightward shift. After enhancement, the ring wall but not the center, exhibited signal intensification **(C,D)**. Twelve hours after hospitalization, the oedema in the lesion-surrounding area became more pronounced; the ventricle displayed severe compression-resulted deformation; and the midline structures showed clear rightward shift **(E)**. Five weeks after the operation, the space-occupying lesion disappeared, but the surrounding area still contained large patches and stripes of low-density shadows with blurred boundary; the surrounding brain tissue showed swelling, with outward bulging in few pockets; the left ventricle displayed enlargement; and the midline structures stayed in the middle **(F)**.

**FIGURE 3:**
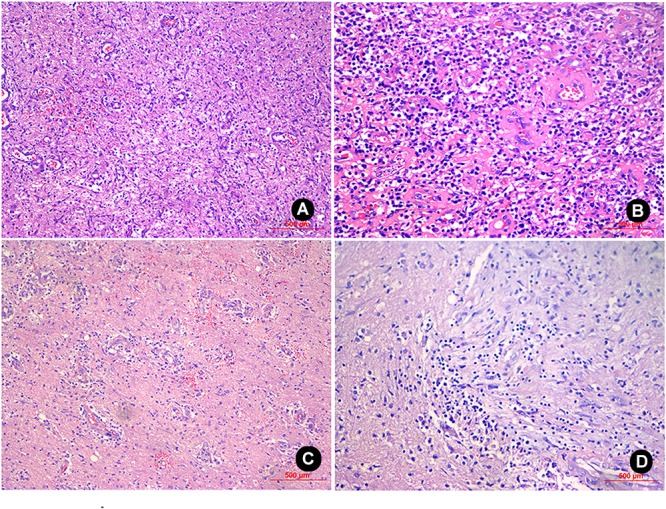
H&E staining of the sample from patient No. 1 (100X and 200X magnification) revealed inflammatory exudates, necrosis, and granulations in the glial cells, and local foci contained mucoid degeneration and micro-abscesses **(A,B)**. H&E staining of the sample from patient No. 2 (100X and 200X magnification) revealed the presence of inflammatory exudates, necrosis, and abscess; the glial cells contained a high level of lymphocyte infiltration and angiogenesis **(C,D)**.

Patient No. 2 was a 45-year-old female with no clear history of recent infection or a past record of sinusitis or otitis media. The patient was hospitalized due to symptoms of headache and right limb weakness for 1 week. A head MRI, including plain and enhancement scans, showed the following: (i) an aberrant, circular space-occupying lesion with long T1 and long T2 signal shadows was observed in the left basal ganglia; (ii) a ring wall with a relatively even thickness presented as slightly short T1 and T2 signals; (iii) the shadow displayed apparent intensification after enhancement; (iv) large patches of long T1 and long T2 oedema shadows were present in the surrounding area of the lesion; (v) the left ventricle exhibited slight compression-resulted deformation; and (vi) the midline structures were shifted lightly toward the left (Figures 2A–D). The patient was suspected of glioma at the local hospital and was transferred to our hospital at 9:00 AM the following day. Upon admission, the patient had a body temperature of 36.3°C, severe headache, and apparent agitation. A physical examination at the Department of Neurology revealed dementia, apathy, a GCS score of 10, babbling, soft neck, a lack of resistance, level 3 right limb muscle strength, normal muscular tension, positive Babinski signs. A complete blood count revealed a leukocyte count of 7.5 × 10^9^/L, 72.7% neutrophils, 19.6% lymphocytes, and CRP level of 18 mg/L. A cardiac echo reminded the heart that everything was normal. After hospitalization, the patient received an intravenous infusion of 20% mannitol 250 ml q8h and was prepared for relevant preoperative examinations. Twelve hours after hospitalization, the patient fell unconscious and had a GCS score of 6, enlargement of the left pupil, loss of light response, avoidance of the left limbs upon needling, and slight buckling of the right limbs upon needling. An emergency head CT showed a spheroid shadow of slightly low density present in the left basal ganglia, the surrounding area had large patches of low-density oedema shadows, the left ventricle displayed severe compression-resulted deformation, and the midline structures exhibited a prominent rightward shift (Figure 2E). The patient was suspected of developing cerebral palsy and thus underwent emergency surgery including lesion resection in the left basal ganglia, resection of the polus temporalis, and a decompressive craniotomy. During the operation, the intracranial pressure was relatively high. As such, the left side of the polus temporalis was first excised, which revealed yellow brain tissue surrounding the abscess and a dark yellow and brittle capsule. The lesion was excised completely after aspiration and decompression. A pathological examination revealed that peripheral lymphocytes infiltrated into the surrounding blood vessels and that some areas were necrotic and accompanied by abscess (Figures 3C,D). After the operation, the patient displayed persistent fever that fluctuated around 38.5°C. A complete blood count revealed a white blood cell count of 7.2 × 10^9^/L, 84.8% neutrophils, 8.9% lymphocytes, and CRP level of 24 mg/L. Pus culture indicated group B *Streptococcus*. Based on a drug sensitivity test, the patient was treated with a regimen of 1 g vancomycin q12h for 3 weeks, after which her body temperature returned to normal, and therapy was continued for an additional 2 weeks. Head CT revealed that the left space-occupying lesion disappeared; large patches or stripes of low-density shadows with blurred boundary were present in the surrounding areas; the surrounding brain tissue displayed swelling; and the midline structures remained in the middle (Figure 2F). However, the patient remained unconscious and was therefore provided with rehabilitation therapy including hyperbaric oxygen treatment for 6 weeks, after which she gradually gained consciousness. Physical examination upon discharge revealed a normal body temperature, high level of consciousness, babbling, level 1 right limb muscle strength, and normal left limb muscle strength.

## Discussion

Brain abscess is a severe, life-threatening infection. It is often found in young and middle-aged individuals, most of whom are male (the male/female ratio is [1.5–3.4]/1) (Lu et al., 2002; Nathoo et al., 2011). Its most common manifestations include headache (∼76% of the patients), fever (∼51%), and focal cerebral signs (∼28%); 5% of patients concurrently develop all three symptoms; 28% of patients suffer from an epileptic attack (Zhang et al., 2014). Its common sources of infection include otogenous, odontogenic, cardiogenic, post-traumatic, haematogenous or secondary-to-pulmonary infections, urinary tract infections, intracranial and meningeal lesions, and skull osteomyelitis. However, some patients with brain abscess carry insidious sources of infection that cannot be identified; these cases are termed “cryptogenic brain abscess.” These patients have no clear history of infection or positive physical signs, which lead to great difficulties in diagnosis. In addition, with the improvement of the prevention and treatment of suppurative otitis media and the extensive use of antibiotics, the brain abscess with a clear source of infection is gradually reduced, and the cryptogenic brain abscess is gradually increasing. Reports showed that the proportion of crytogenic cases accounted for 19.2% of brain abscess cases in 2000 (Roche et al., 2003) and 48% in 2014 (Zhang et al., 2014).

The two patients in the current study had histories physical health and normal immunological function; patient 1 had no record of chronic otitis media or sinusitis, no definitive foci of infection, and no history of head trauma. Patient 2 had only the symptom of headache, and a laboratory examination did not find elevated levels of white blood cells, neutrophils, or CRP.

Based on imaging evidence, brain abscesses can be divided into three stages. The first is the meningoencephalitis stage, which manifests low-density areas with blurred boundaries and uneven density on CT imaging, low-intensity signals with blurred boundaries on MRI T1W1, and high-intensity patches of signals on T2W1; the lesion blends into the surrounding oedema area and displays irregular intensification after enhancement. The second is the suppuration stage, which manifests low-density areas with uneven density, no ring shadows, and apparent oedema in the surrounding area. This stage can lead to a shift of the midline structures, and the lesion manifests irregular intensification after enhancement. The third is the capsule stage, which manifests as even, low-density areas in the center and circular high-density shadows in the surrounding area; after enhancement, the circular shadow displays an apparent intensification with an even thickness and intact, regular contours; the lesion manifests an iso-intense or slight hyper-intense signal on MRI T1W1 and a hyper-intense signal on T2W1. After enhancement, it manifests as a circular intensification that is intact with a thin and even thickness (Nathoo et al., 2011). These manifestations are similar to brain gliomas and metastases; furthermore, they cannot be used as distinct sign for diagnosing brain abscess. The development of MRI technologies has ushered in several imaging methods, including magnetic resonance perfusion imaging, magnetic resonance spectroscopy (MRS), and DWI. MRS enables the accurate identification of brain abscess and cystic/necrotic brain malignancies. For the capsule stage of brain abscess, the necrotic center lacks the metabolites of normal brain tissue (e.g., *N*-acetylaspartic acid, choline, and cholic acid) but has elevated levels of cytoplasmic amino acids and lactic acid. In addition, some patients display concurrent elevations in acetic acid and succinic acid. These are classical resonance signs of an abscess cavity. Although lactic acid and lipid signals can be detected in cases of brain abscess and brain tumor, valine, leucine, and isoleucine are the key markers for diagnosing brain abscess. However, the absence of the three amino acids does not preclude the presence of brain abscess (Pal et al., 2010; Hsu et al., 2013). These traits provide evidence for distinguishing brain abscess and necrotic brain malignancy. DWI is the most valuable method to diagnose brain abscess because it exhibits high sensitivity and specificity when differentiating brain abscess from brain glioma and brain metastases. An abscess cavity manifests as a hyper-intense signal on DWI and a low apparent diffusion coefficient (ADC). By contrast, a necrotic, cystic degeneration area of brain tumor displays hypo-intense signals on DWI and high ADC values (Brouwer et al., 2014). As such, DWI can provide crucial evidence to diagnose and distinguish brain abscess.

Patient No. 1 had a slow disease progression. Prior to operation, head MRI + DWI scans were performed to examine his intracranial conditions. The head MRI showed that the cystic cavity manifested as hypo-intense signals on T1W1 and hyper-intense signals on T2W1, and the surrounding area contained oedema. After enhancement, the lesion manifested as ring intensification; the capsule was intact and had a thin wall of even thickness. DWI indicated that the cystic cavity produced hyper-intense signals, a finding that was consistent with the manifestations of abscess during the capsule stage. Patient No. 2 showed a rapid disease progression. An MRI examination conducted at another hospital indicated an intracranial space-occupying lesion in the basal ganglia. Twelve hours after hospitalization, the patient developed hernia cerebri; thus, head DWI or MRS could not be performed.

The therapies for brain abscess include medication and surgery. Treatment via medication is suitable only during the early suppuration stage, for small abscesses (diameter below 2.5 cm), no apparent increase in intracranial pressure, or multiple brain abscesses without apparent space-occupation (Arlotti et al., 2010). It is recommended that antimicrobial drugs be promptly administered. Gutiérrezcuadra et al. (2009) recommended that the interval between two administrations be no more than 2 days. Before the drug sensitivity test is available, empirical medication may be administered after accounting for the infection factor and lesion site. Antibiotics that are effective and can pass through the blood-brain barrier should be used (Al Masalma et al., 2009). In cases of brain abscess resulting from polybacterial infection at neighboring sites, the administration of cephalosporin combined with metronidazole can be provided. For patients whose contradictions include cephalosporin or metronidazole, meropenem can be used as an alternative (Brouwer et al., 2014). During medication, periodic CT or MRI examinations should be performed to monitor the morphological changes of the brain abscess to evaluate the therapeutic outcome. Signs of effective medication include improvements in clinical symptoms, the alleviation or disappearance of oedema areas based on imaging data, and shrinkage or disappearance of the lesion. At this point, consolidation treatment should be provided for at least 2 weeks. The surgical approaches for brain abscess include puncture aspiration and stereotactic resection. Puncture aspiration is suitable for patients who are not fit for craniotomy because the abscesses have a large lesion size (maximal abscess diameter > 2.5 cm), thin capsule wall, deep location, reside in key functional areas, or because the patients are old, weak, or have other severe diseases. On the other hand, stereotactic resection works better than abscess aspiration for patients who have intact capsule, shallow capsule location, an abscess that does not reside in an important functional area, a thick capsule wall, multiloculated lesions, or patients with cerebral hernia (Brouwer et al., 2014).

Patient No. 1 had an abscess with an intact capsule. The abscess was on the surface of the brain tissue and not in an important functional area, and the capsule wall was relatively thick. Based on these findings, this patient received stereotactic resection, during which surgery towels were used to protect the surgical field. Because of the high brain pressure, approximately 20 ml of yellow, viscous pus was aspirated, with care taken to avoid spillage. After the brain pressure was relieved, the abscess was completely excised along the capsule edge. Patient No. 2 had an acute onset. Twelve hours after hospitalization, her condition worsened, and she became unconscious and exhibited other symptoms (e.g., the formation of cerebral palsy). Emergency surgery was performed including the resection of lesions in the left basal ganglia, the resection of the polus temporalis, and a decompressive craniotomy. Because of the relatively high brain pressure, the left side of the polus temporalis was first removed, followed by an apparent pressure reduction. Lesion resection was then performed. The brain tissue surrounding the lesion was clearly yellow, suggesting the possibility of brain abscess. Diagnostic puncture allowed the aspiration of approximately 10 ml of yellow, viscous pus; subsequently, the patient was diagnosed with brain abscess. The lesion was completely excised along the capsule edge, during which surgery towels were used protect the exposed tissue.

The postoperative bacterial culture of either patient indicated a single microbe of *Streptococcus*. Lakshmi et al. (2011) reported that the pathogens of cryptogenic brain abscess mostly originate from the upper respiratory tract or oral microbiota and are composed primarily of *Streptococcus* spp. and anaerobic cocci. Nathoo et al. (2011) reported that 53.2% of brain abscess cases are caused by a single pathogen of bacteria or fungi, most prevalent agents being *Staphylococcus* (21.2%) and *Streptococcus* (20.2%). These bacteria are often found in post-neurosurgery patients or patients with sinusitis or otitis media. Postoperative patients with brain abscess should receive intravenous antibiotics for 4–6 weeks. In principle, the administration of antibiotics should be maintained until the postoperative body temperature has remained at a normal level for 10–14 days. At this point, follow-up examination using CT or MRI should be performed. For patients with cystic space-occupying lesions, DWI is recommended. If the abscess cavity remains or DWI indicates that the abscess cavity is not close to the cerebrospinal fluid, then the intravenous administration of antibiotics should be continued, even when the body temperature is normal (Arlotti et al., 2010). In this study, the two patients underwent follow-up CT imaging after their body temperatures returned to normal. These scans showed that the cystic space-occupying lesions had disappeared (Figures 1H, 2F).

## Conclusion

In summary, cryptogenic brain abscess is rare in people with normal immune function. However, when a patient develops the classical triad of fever, headache, and focal neurologic deficits, close attention should be paid to his or her disease history and physical examination results, after which the possibility of brain abscess should be investigated. Although we are currently equipped with the most advanced imaging techniques, a large arsenal of antibiotics, and many surgical options, brain abscess remains a disease associated with a high mortality rate. As such, early diagnosis and treatment are crucial to minimize various complications and the number of deaths.

## Author Contributions

WZ and XJ wrote the article. XS collected the materials.

## Conflict of Interest Statement

The authors declare that the research was conducted in the absence of any commercial or financial relationships that could be construed as a potential conflict of interest.
